# Microvesicles as Emerging Biomarkers and Therapeutic Targets in Cardiometabolic Diseases

**DOI:** 10.1016/j.gpb.2017.03.006

**Published:** 2018-02-17

**Authors:** Yan Chen, Guangping Li, Ming-Lin Liu

**Affiliations:** 1 Tianjin Key Laboratory of Ionic-Molecular Function of Cardiovascular Disease, Department of Cardiology, Tianjin Institute of Cardiology, Second Hospital of Tianjin Medical University, Tianjin 300211, China; 2 Section of Endocrinology, Diabetes and Metabolism, Department of Medicine, Temple University School of Medicine, Philadelphia, PA 19140, USA; 3 Department of Dermatology, Perelman School of Medicine, University of Pennsylvania, Philadelphia, PA 19140, USA; 4 Philadelphia VA Medical Center, Philadelphia, PA 19140, USA

**Keywords:** Microvesicle, Microparticle, Exosome, Cardiometabolic disease, Biomarker

## Abstract

**Microvesicles** (MVs, also known as **microparticles**) are small vesicles that originate from plasma membrane of almost all eukaryotic cells during apoptosis or activation. MVs can serve as extracellular vehicles to transport bioactive molecules from their parental cells to recipient target cells, thereby serving as novel mediators for intercellular communication. Importantly, more and more evidence indicates that MVs could play important roles in early pathogenesis and subsequent progression of cardiovascular and metabolic diseases. Elevated plasma concentrations of MVs, originating from red blood cells, leukocytes, platelets, or other organs and tissues, have been reported in various **cardiometabolic diseases**. Circulating MVs could serve as potential **biomarkers** for disease diagnosis or therapeutic monitoring. In this review, we summarized recently-published studies in the field and discussed the role of MVs in the pathogenesis of cardiometabolic diseases. The emerging values of MVs that serve as biomarker for non-invasive diagnosis and prognosis, as well as their roles as novel therapeutic targets in cardiometabolic diseases, were also described.

## Introduction

Microvesicles (MVs, also known as microparticles) were first described in 1967 [1]. At that time, MVs were thought as “dust” from platelets. In the past decades, increasing evidence indicates the importance of MVs in the pathogenesis of various human diseases. MVs are small membrane fragments shed from almost all eukaryotic cells during activation or apoptosis [2], [3], [4]. MVs have been detected in blood, urine, synovial fluid, and many other body fluids from patients with various diseases [5]. Moreover, elevated MV concentrations have already been observed in atherosclerotic plaques, tumor tissue, and other solid tissue/organs in disease states [6].

Growing evidence [7], [8], [9] demonstrates that MVs can harbor not only membrane proteins and lipids from the cell surface, but also nucleic acids (DNA and RNA) including mRNAs, microRNAs (miRNAs), small-interfering RNAs (siRNAs), and long non-coding RNAs (lncRNAs) from the intracellular environment. Because they harbor various components from their parental cells, MVs could perform various functions in intercellular communication, signal transduction, and immune regulation. Therefore, certain specific MVs with some pathological features might be utilized to identify or detect pathological conditions during the development of human diseases. Several studies have indicated that MVs released from blood cells could have potential diagnostic roles in acute coronary syndrome (ACS) [10], ST-elevation myocardial infarction (MI) [11], [12], cardiac remodeling [13], type 2 diabetic mellitus [14], [15], diabetic retinopathy [16], [17], as well as other cardiometabolic diseases. In this review, we provide an overview on the advances of MV-related investigations in cardiometabolic diseases and illustrate the potential roles of MVs as biomarkers or therapeutic targets.

## Characteristics of MVs

**Figure 1:**
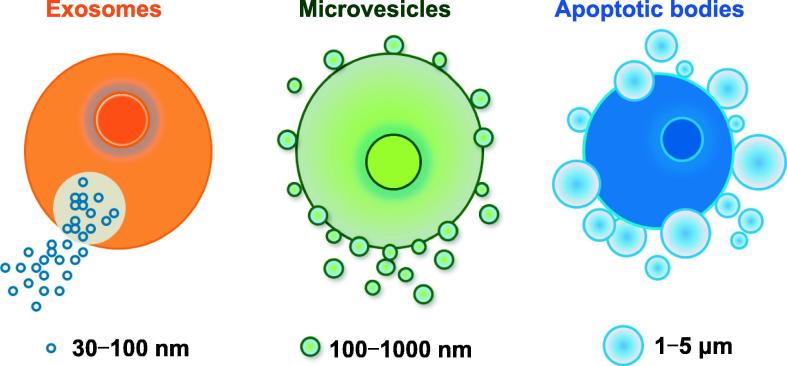
**Extracellular vesicles** Schematic depiction of the extracellular vesicles, including exosomes, microvesicles, and apoptotic bodies. Exosomes are smaller luminal vesicles (30–100 nm in diameter) originating from intracellular endosomes. Microvesicles (also called microparticles) are small membrane vesicles (0.1–1 μm in diameter) released from cell membrane surface during activation or apoptosis of all eukaryotic cells. Apoptotic bodies (1–5 µm in diameter) are released from cell membrane surface in late stage of apoptosis of all cell types.

MVs (0.1–1 μm in diameter), which are budded as small membrane protrusions around a small portion of cytoplasm, could be released from cell membrane surface to extracellular milieu through calpain activation, calcium influx, and cytoskeleton reorganization [18]. In contrast, exosomes are smaller (30–100 nm) and originated from endosomal vesicles through secretion from intracellular luminal space [2]. The extensive plasma membrane budding during apoptotic blebbing forms MVs and apoptotic bodies [19], which have much larger sizes (1–5 μm). Many studies in the literature have indicated differential molecular expression between MVs and exosomes [20]. However, the new term, extracellular vesicle (EV), which comprises exosome, MV, and apoptotic body (Figure 1), has been widely accepted in the field [19], [20], [21], [22].

The most important feature of MVs is heterogeneity. MVs induced by different stimuli could carry different components even if they are from the same cell type. In addition, when different cell types are treated with the same stimulus, the released MVs may also carry different components because of the intrinsic dissimilarity [23]. In fact, some MVs may harbor specific biomarkers from cell surface of their parental cells. Therefore, detecting specific biomarkers on MVs could be used to identify their cellular origins.

In normal cells, anionic phospholipids, such as phosphatidylserine (PS), are only located in the inner leaflet of cell membrane bilayer [24]. During cell apoptosis or release of MVs, PS is shifted to the outer leaflet of the bilayer membrane, and then released with MVs from apoptotic or activated cells [25]. The triggers for the release of cellular MVs include many physical and chemical stimuli, such as cytokines, cholesterol enrichment [26], thrombin, cytotoxic chemotherapy [27], tobacco smoke exposure [28], [29], [30], hypoxia [31], shear stress [32], and many more [19]. Moreover, many of these triggers, *e.g.*, hypertension, atherosclerosis, MI, diabetes, obesity, and hypercholesteremia, are involved in cardiovascular and metabolic diseases [19], [23]. Thus, the release of MVs could reflect the pathological conditions of the disease development. By harboring various components from their parental cells of different origins, MVs, therefore, may bridge the connection between distant cells and play a novel role in intercellular communication during the development of human diseases [33].

### MVs as intercellular messengers

**Figure 2:**
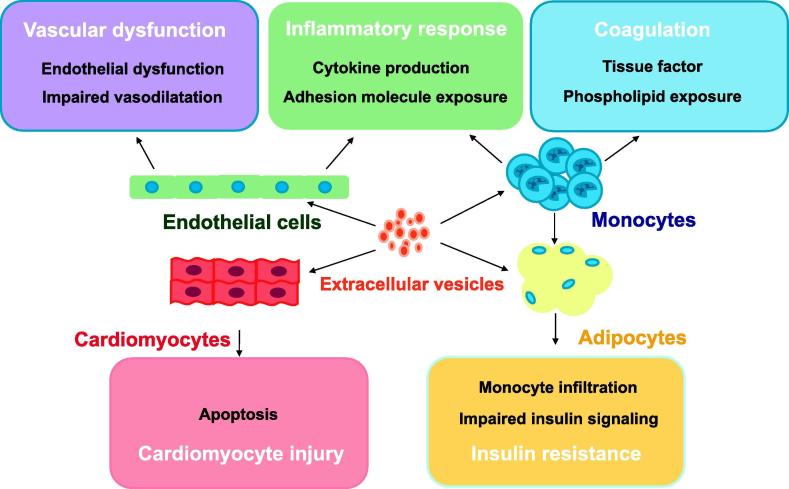
**Mechanisms underlying the involvement of extracellular vesicles in pathologies of cardiometabolic diseases** The potential pathogenic mechanisms of microvesicles underlying coagulation, vascular dysfunction, inflammation, insulin resistance, and cardiomyocyte injury are illustrated. The exposure of EVs to tissue factor and phosphatidylserine could be involved in coagulation cascade and increase the risk of thrombosis in cardiovascular disease and diabetic complications. EVs derived from apoptotic cells have proinflammatory properties by inducing the exposure of adhesion molecules to plasma membrane and secretion of cytokines that are important in atherogenesis and adipose inflammation, thereby contributing to pathogenesis of cardiometabolic diseases. EVs could also directly impair insulin signaling pathways, thus contributing to insulin resistance and metabolic syndrome. The mechanism of vascular dysfunction induced by EVs could be associated with the decreased production of nitric oxide and prostacyclin in the endothelial cells. In addition, EVs could directly affect cardiomyocytes by inducing apoptosis, therefore maybe involved in cardiomyocyte injury and heart damage. EV, extracellular vesicle.

When MVs are associated with specific receptors, the recipient cells could be activated by endocytosis pathway or directly diffuse to the plasma membrane [19]. Therefore, the bioactive components bearing on MVs may participate and mediate thrombosis, endothelial dysfunction, or angiogenesis in the pathological process of cardiometabolic diseases (Figure 2). Due to the exposure of PS and tissue factor (TF), MVs may induce intercellular communication and crosstalk in vascular inflammation and venous thromboembolism [19], [34]. Our recent published work has shown that MVs carry active disintegrin and metalloproteinase domain-containing proteins (ADAMs), in the intraluminal thrombus, close to the aneurysmal wall of abdominal aortic aneurysm (AAA), therefore possibly contributing to the degradation of extracellular matrix of the aortic wall and the development of human AAA [35]. Another work from our group indicates that the nuclear high mobility group box-1 protein (HMGB1) may redistribute from the nucleus to the cytoplasm and plasma membrane of macrophages, and then release to the extracellular milieu with membrane MVs, after exposure to tobacco smoke extract (TSE) [30]. Importantly, these HMGB1-associated MVs may mediate sterile inflammation of various metabolic and autoimmune diseases [19], [30]. In addition, platelet and tumor cell-derived MVs could play a vital role in angiogenesis by transferring a series of proangiogenic factors, including growth factors such as vascular endothelial growth factor (VEGF), basic fibroblast growth factor (bFGF) and platelet-derived growth factor (PDGF), chemokine receptors such as C–C chemokine receptor type 5 (CCR5), C-X-C chemokine receptor type 4 (CXCR4), and matrix metalloproteinases (MMPs) such as MMP2 and MMP9, which contribute to vessel repair, sprouting, and invasiveness [36], [37].

In addition to proteins and lipids, extracellular MVs may also carry DNA, mRNAs, miRNAs, lncRNAs, and other genomic structures [19]. Therefore, MVs may contribute to the transient or persistent phenotypic changes due to their function of transferring genetic information to recipient cells [38], [39], [40]. Extracellular miRNAs packaged by MVs have been shown to play an important role in inflammatory, cardiovascular, and metabolic diseases [41], [42], [43]. For instance, *in vivo* intravascular injection of endothelial cell-derived MVs (EMVs) containing miR-126 can accelerate re-endothelialization in carotid arteries after the mice were subjected to the injury from electric denudation [44]. Let-7 miRNA family was secreted with MVs in metastatic gastric cancer, which may facilitate oncogenesis or metastasis [40]. In addition, Murakami et al. [45] collected human urinary EMVs from healthy donors, and quantified some kidney-specific mRNAs, including *PDCN* and *NPHN* (involved in glomerular filtration), *SLC12A1* (involved in tubular absorption), *ALB* and *UMOD* (involved in tubular secretion), and *AQP2* (involved in collecting duct water absorption). Yang et al. [46] confirmed that MVs were associated with tumor cell-specific human *GAPDH* mRNA in xenografted model of human lung cancer. These studies suggest that miRNAs carried in MVs might play an important role in transferring gene regulation functions.

### Microvesicles as biomarkers of cardiovascular and metabolic diseases

In the past decades, gigantic efforts have been made to identify new biomarkers for detecting the disease risk and monitoring the therapeutic efficacy for the treatment of cardiovascular and metabolic diseases. Among a number of candidates, cellular MVs could serve as biomarkers to provide a comprehensive assessment of certain cardiometabolic diseases (Table 1), although the specificity of MVs is still uncertain.

**Table 1:** Microvesicles as biomarkers in cardiometabolic diseases

**Cell origin of MVs**	**Biomarker for cardiometabolic disease**	**Specific component associated**	**Effects of MVs or mechanisms associated**
Platelet	Atherosclerosis [53], [54] Diabetes mellitus [58], [59], [118], [15] Coronary artery disease [56] Acute coronary syndrome [51] Coronary artery calcification [96] Hypertension [133]	Tissue factor Factor V_a_, VIII_a_, IX_a_ P-selectin GP II_b_/III_a_ CD36	Initiation of coagulation Platelet activation, thrombosis Oxidative stress

Endothelium	Coronary artery disease [60], [65], [66] Ischemic left ventricular dysfunction [61] Obesity [62] Diabetes mellitus [63], [111], [114], [120], [121], [122] Renal failure [64] SLE [67] Hypercholesterolaemia [92] Myocardial infarction [94], [97] Heart failure [103], [104], [105] Diabetic nepheropathy [115], [116], [117] Hypertension [134], [135]	Tissue factor Thromboxane B2 endothelin-1 VCAM-1 RANTES DPP-IV NADP oxidase AGE CD144	Endothelial dysfunction Angiogenesis

Leukocyte	Atherothrombosis [29], [35], [71] Plaque rupture [68], [69], [70] Human abdominal aortic aneurysms [35] Carotid artery thrombosis [72]	IL-1β ICAM-1 MMP14 ADAM10 ADAM17 P2X7R CD45^+^/CD3^+^ HMGB1	Leukocyte activation Monocyte migration Procoagulant activation Thrombosis

Erythrocyte	Sickled cells diseases [73], [74] Diabetes mellitus [112]	Phosphatidylserine	Initiation of coagulation

*Note*: MV, microvesicle; SLE, systemic lupus erythematosus; GP, glycoprotein; VCAM-1, vascular cell adhesion molecule-1; RANTES, regulated upon activation normal T-cell expressed and secreted; DPP-IV, dipeptidyl peptidase IV; NADP, nicotinamide adenine dinucleotide phosphate; AGE, advanced glycation end products; IL-1β, interleukine-1 beta; ICAM-1, intercellular cell adhesion molecule-1; MMP, matrix metalloproteinases; ADAM, a disintegrin and metalloproteinase domain-containing protein; P2X7R, P2X purinoceptor 7; HMGB1, high mobility group box-1 protein.

Various types of MVs are released into the extracellular environment in the human body, carrying membrane, cytosolic, and nuclear components from their parental/donor cells. It is believed that the detectable biomarkers carried or expressed by MVs may be involved in reprogramming of the target/recipient cells [47]. These MVs are easily obtained from body fluids including blood, therefore they can serve as biomarkers in various cardiometabolic diseases [48]. Some studies have shown that the levels of platelet-derived MVs (PMVs) and EMVs are increased in circulation in patients with stable coronary artery disease (CAD) [49], [50], [51], while the levels of leukocyte-derived MVs (LMVs) in the blood are associated with unstable plaques [52].

PMVs are the main culprit in the development of thrombosis, since they provide multiple glycoproteins (GPs) and phospholipids, including PS, GP II_b_/III_a_, and P-selectin. Accumulating studies suggest that PMVs may also contribute to platelet activation during atherothrombosis [53], [54], [55]. Wang et al. [56] have shown that PMV–CD36 complex could activate mitogen-activated protein kinase signaling pathway, mediate oxidative stress and platelet activation in cardiovascular disease (CVD). Furthermore, PMVs might be associated with certain coagulation factors, such as factor V_a_, VIII_a_, and IX_a,_ on the phospholipid membrane surface and result in platelet activation and thrombosis [57]. As expected, circulating PMVs in diabetic patients could be significantly decreased by the antiplatelet drugs taken [58], [59].

Numerous studies have reported that plasma EMVs can serve as a novel biomarker of endothelial dysfunction, suggesting their diagnostic value in several cardiometabolic diseases, such as CAD [60], ischemic left ventricular dysfunction [61], obesity [62], type 2 diabetes mellitus (T2DM) [63], and chronic renal failure [64]. Schiro et al. [65] reported that patients with symptomatic carotid artery disease have significantly elevated plasma levels of endothelial membrane microparticles (EMPs). Compared to the CAD patients with low risk lesions and no thrombi, plasma levels of EMVs are dramatically increased in patients with high risk lesions with multiple irregular thrombi [66]. In patients with systemic lupus erythematosus (SLE), the elevated EMPs levels in blood are associated with endothelial dysfunction. Moreover, immunosuppressive therapy for SLE patients can significantly reduce the circulating EMPs’ levels [67].

LMVs are considered to carry many kinds of bioactive proteins, including interleukine-1 beta (IL-1β), intercellular cell adhesion molecule-1 (ICAM-1), and MMPs. LMVs may be involved in leukocyte activation and monocyte migration to endothelial cells [68], [69], [70]. Furthermore, these extracellular vesicles may play an important role in both atherogenesis and plaque instability [19]. Our previous work has shown that monocyte/macrophage cell-derived MVs that are released after TSE treatment or cholesterol enrichment may serve as novel carriers of danger signals [30], [71], novel contributors to atherosclerosis, immunologic and thrombotic responses *in vivo* [29], [30], [35], [71]. MVs released from macrophages exposed to TSE contain transmembrane protease MMP14 with gelatinolytic and collagenolytic activities [29]. In the intraluminal thrombus of aorta of the patients with AAA, MV-associated active a disintegrin and metalloproteases (ADAMs) may contribute to the degradation of extracellular matrix of the aortic wall during the development of AAA [34]. Furlan-Freguia et al. demonstrated that P2X purinoceptor 7 (P2X7R) stimulated by ATP can increase the release of MVs from macrophages and these MVs may be involved in procoagulation status of carotid artery thrombosis model [72].

In sickle cell disease, erythrocyte-derived MVs aggravate the loss of sickled cells and promote activation of the coagulation cascade [73], [74]. In addition, the presence of genetic components (for example, miRNAs, siRNAs, and lncRNAs) in MVs from blood or other biological fluids suggests that MVs could be considered as novel biomarkers for pathological processes. Therefore, MVs in blood or other body fluids could be exploited in liquid biopsy for diagnostic and prognostic goals [75].

Liquid biopsy has attracted growing attention because the application of this skill has been generalized in detecting tumors, assessing disease prognosis, monitoring systemic treatment methods, and identifying precise therapeutic targets [76]. Currently, three approaches are used in liquid biopsy, including circulating tumor cells, cell-free DNA, and exosomes [77]. Both MVs and exosomes derived from the biofluids of patients could harbor disease-specific mRNAs, miRNAs, proteins, or lipids, thus providing a good source for liquid biopsy in tumor and cardiometabolic diseases. An invasive endomyocardial biopsy has been used to predict the diagnosis of myocarditis by combining immunohistochemistry together with histology [78]. Recent studies [7], [79] have shown that MVs from cardiomyocytes may carry informative cargoes, containing proteins, peptides, several classes of RNA molecules, and sometimes DNA, for similar purpose, therefore they could be exploited for liquid biopsy, prognosis, or therapeutic targets of cardiac diseases.

However, there are still great challenges for the development of reliable and efficient methods for the routine analysis of MVs or exosomes. The mechanism of transporting genetic contents from the cellular context into the MVs remains unclear. Therefore, it is not easy to select the optimal measure to analyze MVs in liquid biopsy. Moreover, the quantitative analysis of the genetic contents in MVs from biofluids could not be achieved yet due to the low abundance of MVs in biofluids and potential loss during sample processing. Another issue is that the components of biofluid could be affected by food intake, medications, or other physiologic and pathological factors. Thus, the optimal method of biofluid collection would be likely to depend on intended assays and biomarkers. In conclusion, there are difficulties in applying MVs in clinical practice for the time being.

## MVs in cardiometabolic diseases

### MVs and atherothrombotic disease

Atherosclerosis is caused by the subendothelial retention, or trapping, of cholesterol-rich, apolipoprotein B (apoB)-containing lipoproteins, particularly low-density lipoprotein (LDL) and remnants. These retained lipoproteins become modified, *e.g.*, by local enzymes, and provoke a series of strikingly maladaptive responses that include chronic sterile inflammation. Rupture-prone plaques comprise depositions of cholesterol and inflammatory cells with only a thin fibrous cap over the atherosclerotic lesion [80], [81].

MVs are considered to be pro-thrombotic since they carry TF, which triggers the initiation of extrinsic coagulation pathway, and PS, which provides phospholipid surface for coagulation factors V_a_, VIII, IX_a_, and II_a_ during coagulation process [82]. Our previous studies indicate that simulation of human acute monocytic leukemia cell line (THP-1 cells) or primary human monocyte-derived macrophages with cholesterol loading or TSE exposure causes the release of TF-positive MVs, which exhibits potent procoagulant activities [26], [28], [72]. In addition to TF and PS, MVs may be associated with other bioactive components, such as P-selectin [83], GP II_b_/III_a_ [84], protein disulfide isomerase [85], as well as factor VIII and V_a_ [86], all of which might be involved in atherothrombotic diseases.

In clinical studies, MVs could induce the release of IL-6 and monocyte chemoattractant protein-1 (MCP-1), and the expression of TF in healthy volunteers [87], [88]. Keuren et al. [89] have found that the exposure to stress-induced PMVs could increase the secretion of IL-8, tumor necrosis factor-α (TNF-α), and IL-1β from monocytes and endothelial cells. All of these cytokines could induce upregulation of leukocyte-endothelial adhesion molecules, and cause the adhesion of monocytes to endothelium and the subsequent subendothelial transmigration, thus contributing to atherogenesis. Moreover, the increased apoptosis or activation of leukocytes, smooth muscle cells, and endothelial cells result in the accumulation of MVs [90]. Suades et al. [91] demonstrate the association between circulating MVs and atherosclerosis in patients with familial hypercholesterolaemia (FH). The FH patients had higher overall circulating MV levels, especially EMVs. Furthermore, the levels of CD45^+^/CD3^+^-MVs were elevated in FH patients with subclinical atherosclerotic plaques. Notably, elevated oxygenized low density lipoprotein in FH may induce the release of MVs derived from monocytes [92]. Therefore, the presence of a considerable amount of pro-inflammatory MVs in vascular wall may be important for the development and progression of atherosclerotic plaque.

### MVs and coronary artery disease

CAD, featured by stenosis or obstruction of coronary artery, leads to the occurrence of myocardial ischemia or even infarction. Increasing clinical evidence has indicated the link between elevated plasma MVs and the risk or incidence of CAD [7], [21], [23]. Moreover, MVs may play an important role in the development of atherothrombotic CAD through their proinflammatory effects, including atherogenesis, induction of endothelial dysfunction, thrombus formation, and plaque rupture [7], [21], [23].

Recent studies demonstrated that patients with ACS have higher levels of PMVs as compared to patients with stable angina or other non-CAD controls [93]. Bernal-Mizrachi et al. [51] have shown the elevated EMVs levels in patients with CAD as compared to healthy controls. Moreover, elevated levels of MVs have been observed in patients with MI as compared to those with unstable angina [51]. A correlation has also been reported between PMVs and thromboxane B2, endothelin-1, platelet-activating factor in patients with coronary intermediate lesions [94]. In addition, there is an increase in PMVs after the application of intravenous ultrasound/fractional flow reserve, suggesting that PMVs may be involved in platelet activation and endothelial dysfunction. Furthermore, Jayachandran et al. [95] find that the increased PMVs are associated with early stage of coronary artery calcification in menopausal women with CAD. By isolating coronary artery endothelial cells (CAECs) and collecting EMVs directly from coronary artery plaque [96], Radecke et al. find increased expression of vascular cell adhesion protein 1 (VCAM-1) on CAECs and EMVs in the patients with MI. Other studies have suggested that presence of regulated upon activation normal T-cell expressed and secreted (RANTES), a proinflammatory member of the C–C chemokine family, may have an important role when evaluating restenosis after percutaneous coronary intervention (PCI). However, PMVs are associated with the levels of RANTES, suggesting that it may be useful for monitoring atherosclerotic events after PCI [97], [98]. However, MVs are not necessarily detrimental in the development of CAD. EMVs may have anticoagulant properties by carrying thrombomodulin and endothelial protein C receptor [99], [100].

Several clinical studies have indicated that MVs could serve as novel therapeutic targets in some conventional treatments for CAD. For instance, the amounts of procoagulant MVs are significantly reduced in the patients with acute MI treated by percutaneous transluminal coronary angioplasty (PTCA) and GP II_b_/III_a_ antagonists [49]. Treatment with n-3 fatty acids in MI patients leads to the decreased levels of PMVs and MMVs, which may possibly explain the mechanisms underlying the anti-inflammatory and anti-thrombotic functions of the treatment in clinical practice [101].

### Microvesicles and heart failure

Heart failure (HF) results from the cardiac remodeling post MI and can be caused by stress through various adverse conditions, like CAD, atrial fibrillation, elevated blood pressure, and valvular heart disease. Cardiac remodeling leads to compensatory hypertrophy of cardiomyocytes and myocardial fibrosis.

Analysis of endothelial dysfunction could be useful for assessment of cardiovascular complications of HF, given the growing evidence supporting the occurrence of endothelial dysfunction in HF [102], [103]. Nozaki et al. [104] recruited 169 consecutive HF patients for detection of the circulating EMV levels, and demonstrated that elevated EMVs could provide valuable information for future cardiovascular events in patients with HF status. In addition, circulating MVs in plasma can be quantified serving as biomarkers for diagnosis and therapeutic monitoring in peripartum cardiomyopathy [105] or Churg-Strauss Syndrome-induced cardiomyopathy [106]. Furthermore, the decreased circulating MVs after immunoadsorption treatment may be related to the improvement of endothelial function in patients with chronic dilative cardiomyopathy [107].

The application of left-ventricular assist device (LVAD) in patients with end-stage HF is important for patients who have been waiting for heart transplantation. However, long-term use of LVAD may cause serious stress in endothelial cells, which consequently would lead to the proatherogenic and prothrombotic changes. Ivak et al. [108] has reported that circulating MVs are increased after LVAD implantation in patients with congestive HF. Moreover, significantly higher MV levels have been detected in patients with LVAD implantation who subsequently suffered from adverse events than those detected at the beginning of their LVAD implantation. Nonetheless, Shah et al. [109] have raised doubt about the role of MVs in LVAD pathobiology, as the elevation of PS+ microparticle levels may be associated with adverse clinical events. Therefore, they reckon that the use of MVs for predicting LVAD complications may be immature for routine identification of the patients with LVAD implantation.

Lately, Zhang et al. [110] analyzed the extracellular vesicle protein levels in a study involving 404 patients who were diagnosed as HF at the emergency room. The protein levels of cystatin C, serpinG1, and CD14 in extracellular vesicles were significantly higher in HF patients as compared to those in controls. In this study, the patients with HF were classified into two groups, that is, HF with reduced ejection fraction (HFREF) and HF with preserved ejection fraction (HFPEF). They found that the levels of serpin G1 and CD14 were elevated in patients with HFREF, whereas the patients with HFPEF had higher serpin F2 and lower serpin G1 levels [110]. Moreover, the levels of serpin G1 in extracellular vesicles were significantly different between HF patients with and without MI history. As a result, they concluded that extracellular vesicle levels of CD14, serpin G1, and serpin F2 are associated with the occurrence of HF in patients suspected for acute HF.

### Microvesicles and diabetes mellitus

Diabetes mellitus (DM), particularly T2DM is associated with accelerated development of atherosclerotic disease, which leads to increased morbidity and cardiovascular complications. Type 1 diabetes mellitus (T1DM) patients have been found to possess a higher number of EMVs, PMVs, and total PS-positive MVs [14]. However, the increase in total MVs seems not statistically significant in T2DM patients as compared to the age-matched controls. In contrast, the levels of PS-positive, erythrocyte-derived MVs were significantly elevated in T2DM patients [111]. Furthermore, the elevated MVs from T1DM patients are associated with procoagulant activity, suggesting their correlation with impaired glucose tolerance and homeostasis [112]. However, MVs associated with dipeptidyl peptidase IV (DPP-IV) in T2DM patients may be involved in glucose metabolism, because DPP-IV can positively affect the incretin degradation [113]. The positive correlation between MV-associated DPP-IV and the urinary albumin/creatinine ratio in patients with diabetic nepheropathy has also been demonstrated in other studies [114], [115], [116].

Both *in vitro* and *in vivo* experiments have shown that procoagulant MVs may trigger and propagate coagulation in DM. The positive feedback loop of thrombin, platelet, and MVs may represent new mechanisms underlying hypercoagulability in diabetes [117]. Cimmino et al. [118] have indicated that MVs associated with procoagulant TF are significantly elevated in patients with DM, suggesting that MVs may serve as a novel biomarker for coagulation. EMVs induced by high glucose stimulation could aggravate endothelial dysfunction, cause macrophage transmigration, and induce the expression of adhesion molecules [119]. The underlying mechanism may be associated with increased nicotinamide adenine dinucleotide phosphate (NADP) oxidase activity and higher reactive oxygen species (ROS) levels [120].

In addition to coagulation and endothelial dysfunction, numerous studies have also illustrated that MVs may be involved in angiogenesis in DM. For instance, Tsimerman et al. [17] have reported that MVs may play important roles in angiogenesis and skin healing in patients with DM. When MVs were incubated with human umbilical vein endothelial cells (HUVECs), angiogenesis could be induced, resulting in the formation of stable and branched endothelial networks. Ettelaie et al. [121] have also demonstrated that the TF-associated MVs could be induced by glucose or advanced glycation end products (AGE) in mesangial cells. These MVs could be involved in angiogenesis of microvascular endothelial cells. However, the angiogenic effect of MVs was dismissed in T2DM patients with CAD, while the relevant mechanism remains unclear [17]. In a rat model of T2DM with insulin resistance, MVs were able to induce the expression of VCAM-1 and the production of ROS in the cardiac endothelial cells of rats on long term high-fat diet [122].

A number of studies have demonstrated that the circulating MVs are elevated in T2DM patients with microvascular complications [123], [124] or CVD, including atherosclerosis. Both circulating EMVs and MVs derived from endothelial progenitor cells are elevated in the ischemic stroke models of diabetic db/db mice [125]. In T2DM patients with ACS, circulating EMVs positive for CD144 are associated with unstable coronary plaques [126]. However, in diabetic patients without typical angina symptoms [15], CD144-positive EMVs are the most significant and sensitive biomarkers as compared to the traditional cardiovascular biomarkers. Many studies have also demonstrated that circulating MVs are involved in pathological progression of diabetic retinopathy with retinal vascular occlusion [124], [16], diabetic nepheropathy [127], and diabetic neuropathy [128].

### Microvesicles and other cardiometabolic diseases

Obesity is another metabolic disorder that is considered as the imbalance between energy intake and expenditure, characteristic with hypertrophy and hyperplasia in adipocytes [129]. Our recent work has demonstrated that human macrophages stimulated by TSE may cause the release of MVs with HMGB1 [30], and these HMGB1-positive MVs can impair insulin signaling in cultured adipocytes (unpublished work). In line with our findings, Zhang et al. [130] have reported that MVs derived from proinflammatory macrophages may impair insulin signal transduction, particularly the activation of signaling pathways that cause glucose uptake. Furthermore, MVs released from primary adipocytes, which were associated with numerous complicated components, such as leptin, TNF-α, fibroblast growth factor-γ (FGF-γ), MMP-2, and MMP-5, could induce angiogenesis *in vivo* [131].

Arterial hypertension, which is a vital risk factor for atherosclerosis, stroke, and CAD, has been regard as the consequence of endothelial dysfunction in early stage of these diseases. Preston et al. [132] have demonstrated that both EMVs and PMVs are significantly elevated in severe hypertension, while EMVs were associated with the level of both systolic and diastolic blood pressures. As the final target of the renin angiotensin system, angiotensin II could accelerate the release of prothrombotic MVs from mononuclear cells in patients with arterial hypertension [133]. Hsu et al. [134] report that elevated EMVs may serve as biomarker to assess the impaired kidney functions since the increased EMVs have been associated with the decrease in glomerular filtration rate in patients.

## Microvesicles in therapy of cardiometabolic diseases

Martinez et al. [135] have demonstrated that circulating MVs may be considered as biomarkers to monitor therapeutic efficacy in various cardiometabolic diseases. Cholesterol-lowering statins have been proved to reduce the levels of circulating MVs derived from leukocytes, platelets, and endothelial cells [136], [137]. Similarly, other cardioprotective drugs, including angiotensin II receptor blockers [134], calcium blockers [138], aspirin [139], and clopidogrel [140], have also been shown to reduce blood levels of MVs in patients. However, the underlying mechanisms remain to be elucidated. The beneficial effects of these drugs may either result from their direct effects or simply be the consequences of the decreased cholesterol, inflammation, or overall cardiovascular risk.

### Microvesicles in regenerative therapies

Initial studies regarding the therapeutic MVs from mesenchymal stem/stromal cells (MSCs) have been reported in animal models of acute kidney failure and MI [141]. Increasing evidence [142], [143] has demonstrated that exosomes derived from MSCs have cytoprotective effects on pulmonary hypertension induced by hypoxia, alleviate acute lung injury stimulated by endotoxin, and accelerate muscle regeneration in mice. In addition to immune modulation, MSC-MVs are also used in therapeutics by inducing neurogenesis and angiogenesis [144]. Chen et al. [145] have reported that MSC-MVs could facilitate miRNA-mediated intercellular communication. Exosomes secreted from GATA-4 overexpressing MSCs are cardioprotective by regulating the expression of anti-apoptotic miRNA in recipient cells [146]. In animal model of myocardial ischemia/reperfusion injury, MSC-derived exosomes could prevent adverse remodeling and enhance myocardial viability of the affected mice, through restoring bioenergetics, reducing oxidative stress, and activating pro-survival signaling [147]. Therefore, treatment using MSC-derived exosomes may be a potential adjuvant therapy to reperfusion treatment for MI patients.

Extracellular vesicles released from endothelial colony forming cells (ECFCs) can also stimulate neurogenesis and angiogenesis *in vitro* and *in vivo* [148], [149]. ECFC-MVs have been shown to promote revascularization and protect the kidney function in mouse model with ischaemia/reperfusion injury [150]. Arecent report has suggested that exosomes derived from cardiac progenitor cells could induce transmigration of endothelium and protect myocardial ischemia/reperfusion injury [151]. Although further investigations are still needed to optimize the efficacy, the application of stem cell-derived vesicles might provide novel insights into cardiovascular regenerative therapy in the future.

### Microvesicles in gene therapies

As a potential tool in gene therapy, MVs are able to harbor and transport genetic information to distal target cells. MVs derived from endothelial progenitor cells, which are associated with specific mRNAs, could activate angiogenesis through phosphatidylinositol 3 kinase/protein kinase B signaling pathway [152]. Circulating MVs can shuttle numerous specific miRNAs involved in fundamental signal transduction processes of CVD [153]. For instance, MVs derived from human THP-1 cells treated by inflammatory factors could harbor miR-150 [154], and c-Myb targeted by miR-150 may be involved in endothelial cell migration [155]. Furthermore, miR-150 was elevated in EMVs in blood vessels of mice. Similarly, MVs that carry miR-126 play an important role in angiogenesis and vascular integrity [156], [157]. Notably, application of the miR-126-enriched MVs to ApoE−/− mice could limit the development of aortic plaques of atherosclerosis [158].

### Microvesicles as promising therapeutic delivery tools

As we discussed above, MVs that contain RNA, DNA or proteins may be involved in regulation of signaling pathways during pathological processes. Therefore, gene therapy and specific drug delivery may be achieved in the future by inhibiting the formation, release or delivery of MVs. The advantages of applying MVs in therapeutic delivery include decreased toxicity or immunogenicity and increased stability of intracellular environment [159]. In addition, some studies have indicated that exosomes could serve as anti-cancer drug delivery vehicles [160]. Paclitaxel, an anti-inflammatory drug, has been used to treat MSCs *in vitro* for stimulating the secretion of exosomes. Interestingly, the paclitaxel-incorporating exosomes have been found to restrain tumor cells from proliferation [161]. In addition, exosomes induced by curcumin, a strong inhibitor to tumor cells in the progression of various kinds of cancer, have been demonstrated to block the activation of myeloid cells and subsequently suppress the apoptosis of microglial cells [162], [163]. Although most of the studies above have been conducted in tumor therapy, MVs as a therapeutic modality might provide promising insights for its application in cardiometabolic diseases in the future, as MVs might have similar protective function in cardiometabolic diseases.

## Conclusions

Increasing evidence highlights the contribution of MVs to the pathogenesis and progression of cardiometabolic diseases, including atherosclerosis, stroke, CAD, cardiac hypertrophy, and diabetes. MVs could also serve as novel biomarkers for pathologic conditions, including thrombosis, inflammation, endothelial dysfunction, or angiogenesis, in various cardiometabolic diseases. More and more studies also indicate that the molecular characteristics of MVs and their cellular origins could not only reflect the nature of the diseases itself but also be affected by the progress and the treatments, which may provide powerful tools for diagnosis, prognosis, and drug treatment monitoring. However, our understanding on the contributions of MVs to pathophysiological processes of human diseases and their potential mechanisms are still limited, more novel diagnostic or therapeutic approaches and methods involving MVs are expected. In addition, there are still limitations in clinical application of MVs as biomarkers. Firstly, the standardized methods for blood collection and analysis for MVs have not yet been established. It would be better if more sensitive and specific methods could be established, particularly MVs in other body fluids could also be evaluated in the future. Secondly, the underlying mechanisms of cardiometabolic diseases are complicated, which may affect the accuracy of diagnostic and predictive application of MVs in cardiovascular events. Thus, exploration of specific targets would be the focus of the future investigations. Furthermore, most of the published studies had focused on the cell membrane molecules. Interestingly, we have recently found that cytosolic and nuclear molecules can also be carried by MVs [30]. Therefore, efforts in further exploring the MV-associated cytosolic and nuclear molecules and their roles in cardiometabolic diseases may also be needed.

In conclusion, studies on the molecular mechanisms underlying the formation and release of MVs, as well as their specific functions in cell–cell communication, may lead to new perspectives and therapeutic strategies for improving the outcome of cardiometabolic diseases.

## Competing interests

The authors have declared no competing interests.
